# Application of Carbon Ion and Its Sensitizing Agent in Cancer Therapy: A Systematic Review

**DOI:** 10.3389/fonc.2021.708724

**Published:** 2021-07-05

**Authors:** Xiaolin Wang, Xiaojun Chen, Guangfei Li, Xiao Han, Tianxin Gao, Weifeng Liu, Xiaoying Tang

**Affiliations:** School of Life Science, Institute of Engineering Medicine, Beijing Institute of Technology, Beijing, China

**Keywords:** heavy ion radiotherapy, carbon therapy, radiation therapy, clinical application, sensitizing agent

## Abstract

Carbon ion radiation therapy (CIRT) is the most advanced radiation therapy (RT) available and offers new opportunities to improve cancer treatment and research. CIRT has a unique physical and biological advantage that allow them to kill tumor cells more accurately and intensively. So far, CIRT has been used in almost all types of malignant tumors, and showed good feasibility, safety and acceptable toxicity, indicating that CIRT has a wide range of development and application prospects. In addition, in order to improve the biological effect of CIRT, scientists are also trying to investigate related sensitizing agents to enhance the killing ability of tumor cells, which has attracted extensive attention. In this review, we tried to systematically review the rationale, advantages and problems, the clinical applications and the sensitizing agents of the CIRT. At the same time, the prospects of the CIRT in were prospected. We hope that this review will help researchers interested in CIRT, sensitizing agents, and radiotherapy to understand their magic more systematically and faster, and provide data reference and support for bioanalysis, clinical medicine, radiotherapy, heavy ion therapy, and nanoparticle diagnostics.

## Introduction

Currently, noncommunicable diseases (NCDs) are the leading cause of death globally, and cancer is expected to become the leading cause of death worldwide in the 21st century and the single most important barrier to improving life expectancy (1–3). Cancer has a high incidence and mortality rate worldwide, which is the first or second leading cause of death globally. According to the latest report data of World Health Organization (WHO), worldwide, an estimated 19.3 million new cancer cases occurred in 2020, of which 49.3% in Asia and 22.8% in Europe; almost 10.0 million cancer deaths occurred, with 58.3% in Asia and 19.6% in Europe. The most common cancers were breast, lung, prostate and colon cancer, with breast cancer having the highest incidence at about 11.7%. In addition, the cancers with high mortality mainly included lung cancer, liver cancer, stomach cancer and breast cancer, among which the death rate of lung cancer was the highest, up to 18.0%, followed by liver cancer, which was about 8.3% (4).

Radiation therapy (RT) is one of the oldest forms of cancer treatment, with more than 50% of cancer patients receiving additional RT at various stages, and more than 70% using RT in developed countries (5). More than two-thirds of cancer patients receive RT alone or in combination with other cancer treatments, such as surgery or chemotherapy, especially those with local or regional advanced stage (6). With the rapid development of science and technology, radiotherapy technology has been developing constantly. The common RT mainly includes X-ray radiotherapy (XRT), γ-ray radiotherapy (γRT), electron radiotherapy, proton therapy (PT) and heavy ion radiation therapy (the most common heavy ion therapy is carbon ion therapy, CIRT). X-rays are made up of photons and can pass directly through the body, but they may have serious side effects when they pass through healthy tissue. Compared to X-rays, γ-rays have shorter wavelengths and are more penetrating, and gamma knife currently refers to γ-rays. However, the action of γ-rays is usually relatively slow and the damage to normal tissue is significant. Protons are positively charged particles that stop moving when they hit a target, reducing the chance of causing damage to healthy tissue. Among the new technologies in RT, the use of carbon ions marks a new era in the field of high-precision cancer treatment. Carbon ions have a larger mass, which reduces the transverse scattering of carbon ions and improves the radiation accuracy. On top of that, the dose of carbon ions drops faster than the dose of protons, which keeps the normal tissue around the tumor in better shape. The unique physical and biological characteristics of CIRT give it significant advantages over other RT (7). So far, CIRT has been used in almost all types of malignant tumors and has been extensively studied in recurrent diseases.

Here we summarize CIRT unique physical and biological characteristics and advantages, and lists the clinical trials and research by CIRT in recent years, and summarized the related clinical data, including the number of cases, carbon ion dose, local control rate, over survival rate and the toxicity. Finally, the related radiation sensitizers of carbon ion were preliminarily explored. We believe that CIRT is very promising and has the potential to be the most attractive cancer treatment option.

## The History and Characteristics for CIRT

### History

**Figure 1:**
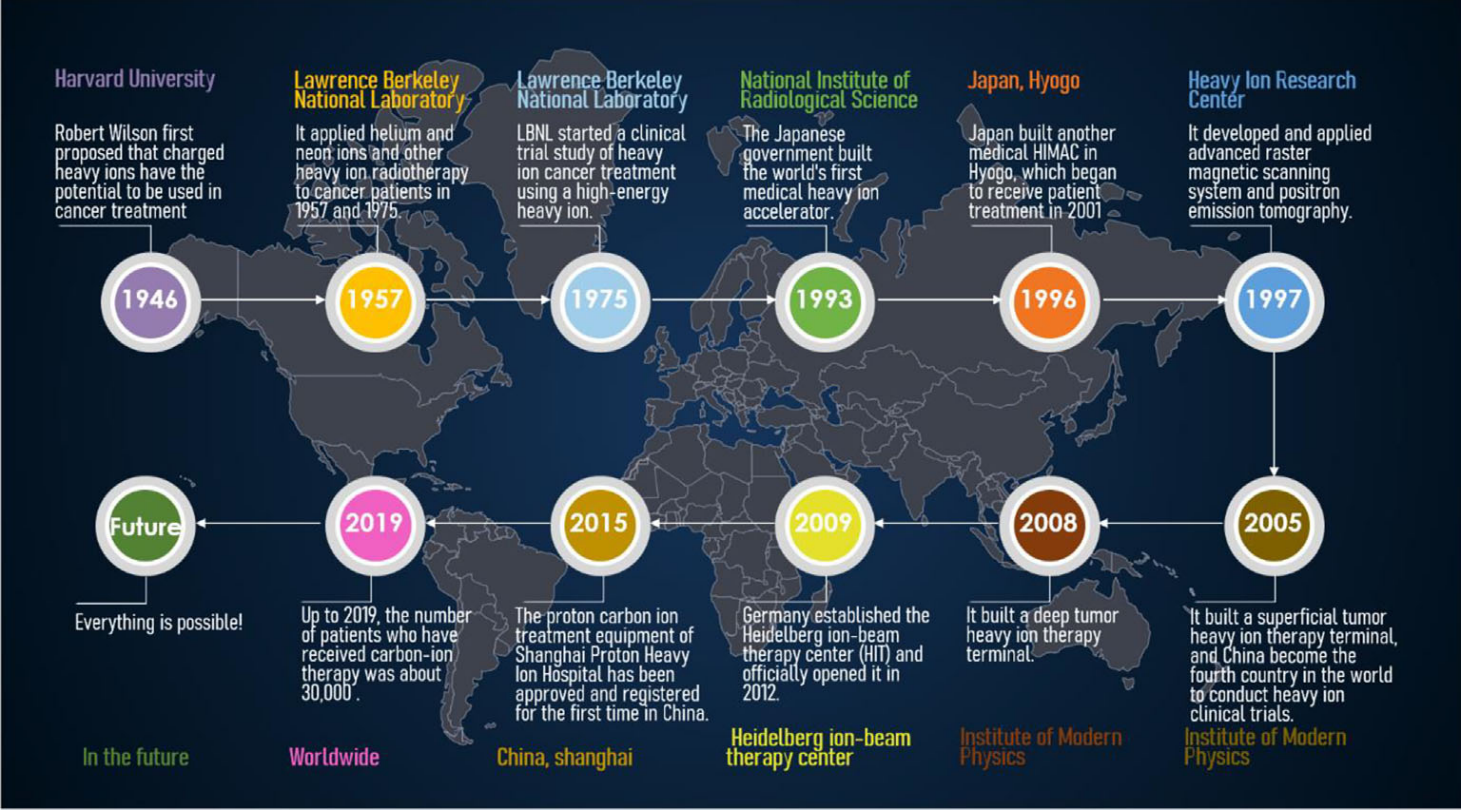
Timeline of major events in heavy ion therapy.

In 1946, Robert Rathbun Wilson was the first to propose the use of heavily charged particles and fast protons for the treatment of cancer. In 1954, Lawrence Berkeley National Laboratory (LBNL) first used protons for therapeutic studies, and helium ions were studied three years later. In 1975, LBNL started a clinical trial study of heavy ion cancer treatment using a high-energy heavy ion synchronous cyclotron. It was found that the local control rate of heavy ion radiotherapy was 2-3 times higher than that of conventional radiation such as X-rays, gamma rays and electron beams. In 1990, the American Fermi Laboratory used a rotating gantry (which can rotate the number of protons) to build the first proton beam radiotherapy equipment. This equipment can emit proton beams from different directions through the isocentric gantry, thereby reducing skin and damage to normal cells between tumors, increasing the scorch-to-skin ratio during treatment. In 1993, the Japanese government built the world’s first heavy ion medical accelerator (HIMAC) at the National Institute of Radiological Science (NIRS) in Chiba Prefecture, which is specially used for CIRT and radio medical research (8). More than twenty thousand patients were subsequently treated with CIRT (9). HIMAC treatment device mainly includes synchrotron, beam distribution and irradiation system, patient positioning system and treatment plan system. Its ion beam type is ^4^He~Ar, beam intensity is 10^7^~10^10^pps, and the maximum energy can reach 800 MeV/u dose rate, and control at about 5 Gy/min. The patients treated included head and neck tumors, brain tumors, lung cancer, liver cancer, prostate cancer, and cervical cancer. For head and neck tumors, a local tumor control rate of more than 80% has been achieved; for overall treatment, good curative effects have been achieved without obvious complications, and the tumor growth inhibition rate is high. Encouraged by the results of this HIMAC treatment, Japan built another medical HIMAC in Hyogo in 1996. The synchrotron can provide 230 MeV proton beam, 230 MeV/u helium ion beam and 320 MeV/u carbon ion beam. The treatment device was completed in 2000 and began to receive patient treatment in 2001. In 1997, the Heavy Ion Research Center in Darmstadt, Germany, used the treatment characteristics and experience of the ^20^Ne ion beam of the American LBNL and the ^12^C ion beam of the Japanese NIRS to develop and apply advanced raster magnetic scanning system and positron emission tomography. The two major technical methods of surgery have achieved heavy ion beam conformal radiotherapy and real-time online monitoring of beam current. In December of the same year, two cases of skull base tumors were treated with heavy ion beams. A clinical follow-up study three months after treatment showed that hypocranial tumors basically disappeared. In 2005, the Institute of Modern Physics (IMP) of the Chinese Academy of Sciences built a superficial tumor heavy ion therapy terminal based on the Lanzhou Heavy Ion Research Facility (HIRFL), and used the 80 MeV/u carbon ion beam provided by HIRFL in the following year. The Multi-Layer Cancer Terminal conducted the first clinical treatment trial on 4 patients with melaleuca malignant cases, which also made China the fourth country in the world to conduct heavy ion clinical trials. In 2007, IMP used 100 MeV/u carbon ion beam provided by HIRFL to treat 23 patients with tumors. After a course of carbon ion radiotherapy in the 27 patients before and after, most of the tumors of the patients have completely disappeared, and the rest of the patients have reduced to varying degrees. The patients did not have any local or systemic adverse reactions. At the end of 2008, IMP built a deep tumor heavy ion therapy terminal based on the Lanzhou HIMAC cooling storage ring. By the end of 2014, a total of 18 batches of 213 tumor patients (103 cases of superficial and 110 cases of deep) were treated before and after IMP, and significant effects were achieved. In 2009, Germany established the Heidelberg ion-beam therapy center (HIT) and officially opened it in 2012. HIT mainly treats brain tumors, thyroid tumors, lung (far away from the heart) tumors, liver tumors, and prostate tumors. In March 2015, after review and approval by the China National Food and Drug Administration (CFDA), the proton carbon ion treatment equipment of the Shanghai Proton Heavy Ion Hospital was approved for the first time in China and officially operated in May of the same year. The main types of diseases currently treated are: nasopharyngeal carcinoma, chordoma, chondrosarcoma, early and locally advanced lung cancer, part of thymic cancer and chest metastatic tumors, liver cancer, pancreatic cancer, prostate cancer, etc. Regardless of whether these cancer patients received treatment during the treatment phase or the discharge follow-up phase, the tumor condition control was good, the patient’s disease indications also stabilized, and the overall condition was good. For the entire timeline of events, kindly refer to **Figure 1**.

**Figure 2:**
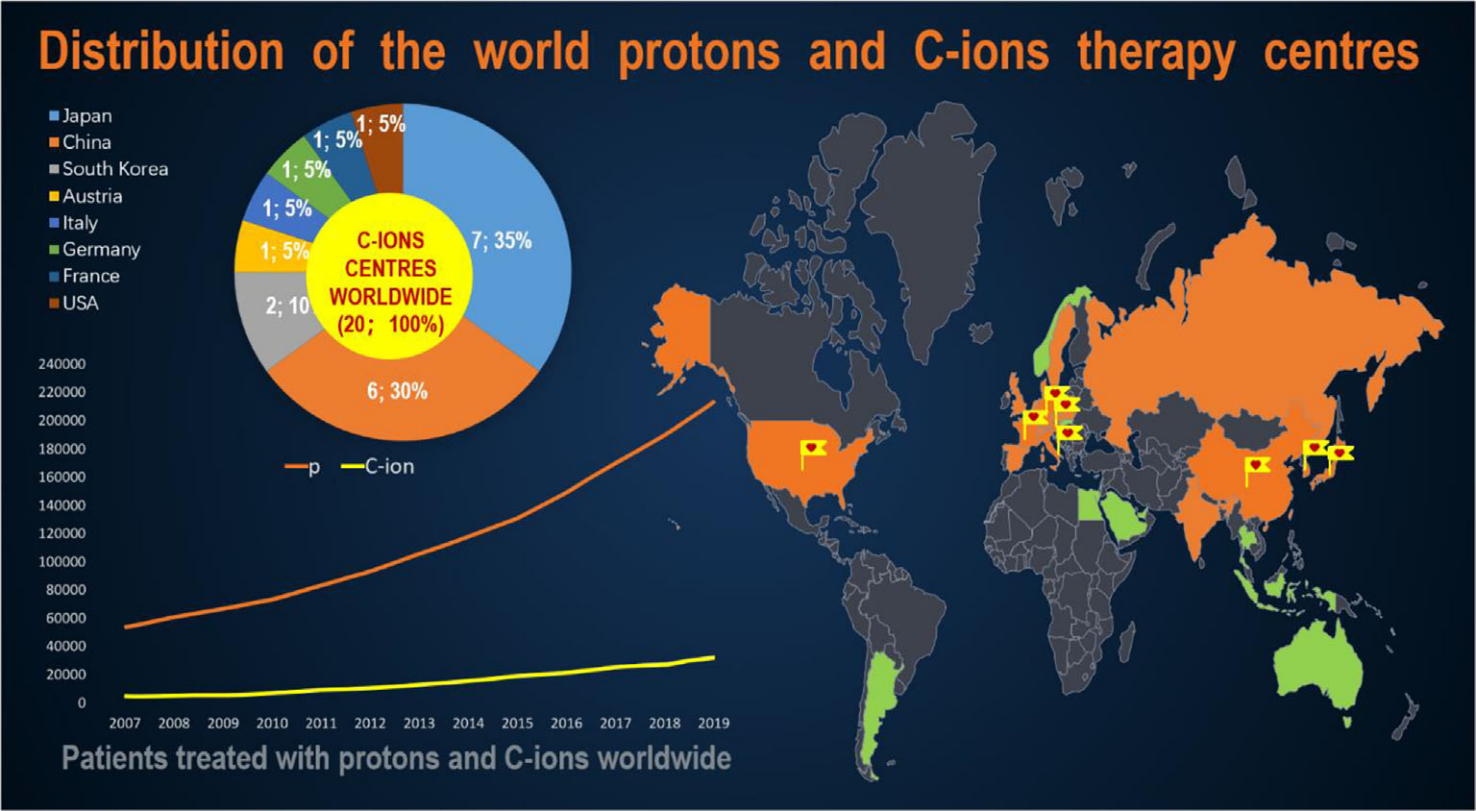
Distribution of the world protons and C-ions therapy centers.

According to the data published by the Proton Therapy Co-Operative Group (PTCOG, https://www.ptcog.ch/, last update: Feb 2021), there are nearly 175 particle therapy centers in operation, construction or planning worldwide, including 12 CIRT centers in operation. The distribution of the world protons and CIRT center in countries of the world and the number of patients who have received particle therapy have been presented in **Figure 2**. In addition, according to the “Patient Statistics” published by PTCOG, we know that about 220,000 patients are treated with particle therapy, and about 14% of them are treated with CIRT. The rapid development of heavy ion radiotherapy is in Japan and Germany. Japan is also the country with the most clinical trials of CIRT (10). Currently, six carbon ion treatment centers are under construction and two are planned. **Table 1** details the number of CIRT centers worldwide, the highest energy, the type of accelerator, the start time of treatment or the planned treatment time. As can be seen from **Table 1**, the use of CIRT is not yet widespread and is still considered “experimental” for many tumor sites, but clinical indication guidelines are being developed (11).

**Table 1:** Distribution of the world C-ions therapy center in operation, under construction and in planning stage.

Operating conditions	Country	Who, Where	MAX. Energy (MeV)	Start of treatment
Facilities in Operation	Austria	MedAustron, Wiener Neustadt	S 403/u	2019
	China	SPHIC, Shanghai	S 430/u	2014
	China	Heavy Ion Cancer Treatment Center, Wuwei, Gansu	S 400/u	2019
	Germany	HIT, Heidelberg	S 430/u	2009, 2012
	Germany	MIT, Marburg	S 430/u	2015
	Italy	CNAO, Pavia	S 480/u	2012
	Japan	HIMAC, Chiba	S 800/u	1994, 2017
	Japan	HIBMC, Hyogo	S 320/u	2002
	Japan	GHMC, Gunma	S 400/u	2010
	Japan	SAGA-HIMAT, Tosu	S 400/u	2013
	Japan	i-Rock Kanagawa Cancer Center, Yokohama	S 430/u	2015
	Japan	Osaka Heavy Ion Therapy Center, Osaka	S 430/u	2018
Facilities under Construction	China	HITFil at IMP, Lanzhou, Gansu	400/u	2021?
	France	ARCHADE, Caen	400/u	2023
	Japan	Yamagata University Hospital, Yamagata	430/u	2021
	South Korea	KIRAMS, Busan	430/u	2025
	South Korea	Yonsei University Hospital, Seoul	430/u,	2022
	China	Taipei Veterans General Hospital, Taipei	430/u	2021/2022
Facilities in Planning Stage	China	Himed Cancer Hospital, Xuzhou City, Jiangsu Province	430/u	2022?
	USA	Mayo Carbon Ion Therapy Center, Jacksonville, FL.	250, 430/u,	2025+

S, Synchrotron; ? = open (last update: Feb 2021).

### Rationale

#### Physics Rationale

In the process of penetrating tissues, charged particles can produce a characteristic depth dose curve. The radiation dose is released rapidly at the end of the particle’s range and reaches the peak, forming the Bragg peak. CIRT often takes advantage of this characteristic to insert Bragg peak into tumor tissue. But because Bragg Peak is usually not wide enough to cover most tumor tissues. The penetration depth is a function of initial kinetic energy and particle charge. A higher energy particle can penetrate deeper, while a larger charged particle with the same initial kinetic energy can penetrate shallower. So using particles with different initial kinetic energy properly weighted, each other can in depth (or bundle) directions to create a uniform dose (or is, in fact, any shape the physical dose distribution) of areas to cover need treatment of lesions, produce a spread-out Bragg peak (SOBP), covering the entire tumor tissue, thus providing the required radiation dose to the target tissue. This dose deposition pattern is the basis of charged particle therapy for malignancy, enhancing dose distribution and transverse focusing. SOBP enables particle therapy to have a good dosimetric distribution, which maximizes the killing of tumor tissues and protects the normal tissues around the tumor to the greatest extent.

This physics rationale of SOBP offer several significant benefits for particle therapy compared to other RT (12): First, the proportion of the dose deposited into the tumor is increased relative to the dose deposited in healthy tissue close to the tumor. Second, less dose is given to normal tissue at the back end of Bragg Peak, which allows more retention of normal tissue at the distal edge of the tumor (13). Third, they can be magnetically guided rather than physically aligned, which allows clinicians to map a three-dimensional tumor with radiation doses while minimizing radiation to nearby dangerous structures. Compared with PT, CIRT shows inhibited multiple coulomb scattering during the movement of carbon ions, which results in a sharper side penumbra of the heavy ion beam. The clinical use of the fact that the carbon ion beam can potentially be placed laterally closer to the organ at risk while maintaining a high degree of organ preservation. On the other hand, because the relative bioavailability (RBE) of PT is only 10% higher than that of XRT (14), therapeutic resistance may still exist in the tumor microenvironment. Carbon ions, on the other hand, take advantage of their greater mass, which leads to more severe DNA damage, and the new effect of this damage can be increased by about 2-4 times (15, 16).

The possibility of dose verification by available imaging. Dose verification is a key link in the process of CIRT, which can accurately evaluate the tumor target area and surrounding normal tissue of patients, improve the target dose and local control rate, and reduce the radiation dose of target area and surrounding normal tissue, so as to ensure the treatment quality of CIRT. As carbon ions move through a substance, nuclear interactions occur in both the ion projectile and the traversing substance. Some of these nuclei interact to produce positron emission nuclei, which can be imaged using positron emission tomography (PET) scanners.

Superior linear energy transfer. CIRT has a potential clinical advantage in that heavily charged particles with higher let values than photons or protons are used. It is widely believed that carbon ions are more effective in the treatment of radiation-resistant cancers, such as recurrent nasopharyngeal cancer, prostate cancer, bone and soft tissue tumors, head and neck cancer and so on, because of their high linear energy transfer (LET), which can cause more direct double-stranded DNA damage (17). The LET of a particle can be determined by many factors, but the two most important factors are ion charge and ion velocity. Heavier charged particles can adjust their speed to meet high or low LET requirements. In the entrance area of tissues, their LET values are lower due to their high speed, and increase under lower kinetic energy as the particles stay in the deeper area of the tumor. Osama et al. (6) believed that the dose distribution of particles with high LET values on the nanometer and micron scales was the result of large ionization event clusters along particle tracks caused by direct or collision events (intensive ionization). This event indicates a sharp increase in trace doses, leading to the destruction of DNA and other related biomolecules, which is considered to be more complex, so it may lead to more serious relative biological effects (RBE) than low LET radiation, which means that the higher the LET value of the same absorbed dose, the greater the RBE (18).

#### Biological Rationale

The biological principles of CIRT mainly include high RBE, more complex DNA damage, higher oxygen enhancement ratio and more complex lethal mechanism. The researchers came up with the concept of RBE in order to compare the effects of different types of radiation. RBE is defined as the ratio of the test radiation dose to the reference radiation that produces the same biological endpoint. X-rays are generally considered to have an RBE of 1, independent of energy. The International Radiation Unit and Measurement Committee recommends that the RBE value of the proton is 1.1 (19). Compared with other rays, carbon ion rays have a higher RBE. The RBE value of carbon ions that is clinically recognized is 1.1-4.0 depending on the cell line (11), but there are also reports in the literature that its RBE value reaches 5. There are many factors that affect the RBE value, such as LET, ion type, dose per fraction, cell/tissue type of action, etc. Therefore, for C ions, RBE value is variable, so more experiments and data are needed to verify. Jeong studied and summarized the relative biological effects of CIRT on early lung cancer (10). In this work, they used a tumor mechanism response model for photonic radiotherapy of the lung to estimate the RBE of CIRT relative to photonic radiotherapy. Fractional dose, number of fractions, treatment schedule, and local control rates were used to simulate the model with respect to the standard photon results. The dose-response relationship of the obtained CIRT was compared with the previously determined dose-response relationship of photonic radiotherapy for lung cancer, and the RBE of the CIRT was derived. The results showed that with the increase of fractional dose, the number of fractional dose decreased, and the RBE value decreased, and the derived RBE ranged drops from 2.1 to 1.5. In this study, the clinical experience of photonic radiotherapy and CIRT in the treatment of early lung tumors was integrated, with few fitting parameters and clear mechanism significance.

Unlike low LET radiation, when using a high-LET CIRT, humans are exposed to a high energy charge depositing a large amount of energy along the through-path. DNA damage caused by high LET radiation is complex and varied, including single strand breaks (SSBs), chemically altered base damage, intra strand crosslinking, double strand breaks (DSBs) and “cluster” damage, etc. The complex DNA damage repair response caused by high LET radiation is less efficient, and what’s more it is difficult to use a single DNA repair method to repair, which may eventually lead to genomic instability and cell death. The damage of complex DNA aggregation induced by high LET radiation (including carbon ions) is caused by multiple DNA damage, and the specific mechanism needs to be further studied. In fact, many reports have confirmed this complexity. The main reason for the increase in cancer mortality is the inability of DNA repair pathways to faithfully handle these repaired or mishandled complex lesions. In addition, since carbon ions with high LET induce complex DNA damage that is difficult to repair, CIRT is considered to be effective in killing chemoradio-resistant tumors.

In addition to the direct damage to DNA caused by carbon ion radiation, carbon ion radiation also affects the liquid in the cell. For example, in the process of radiation, the water in the cell will cause hydrolysis and lead to the accumulation of reactive oxygen species (ROS), and the accumulation of these reactive oxygen species will cause secondary damage to tumor cells, tissues, etc., that is, the so-called “indirect damage”. One requirement for maximum ROS-related damage is the presence of molecular oxygen, which fixes or makes permanent the damage caused by ROS. Quantifying the effect of oxygen is done through the oxygen enhancement ratio (OER). Similar in concept to RBE, the OER seeks to assess the amount of dose necessary to result in an equivalent biological endpoint with or without the presence of oxygen. In contrast, the estimated OER for carbon and other heavy ions may decrease with the increase of LET, and the OER can drop from 2.5 to 1.0 depending upon the ion charge and LET (20).Therefore, high LET particles at the appropriate LET (depths) are more effective at killing cells in the hypoxic, necrotic cores of tumors compared to photons, lending particle therapy yet another biological advantage over photons. In addition, heavy ion scattering is relatively small, which is very beneficial to accurate dose distribution.

CIRT has little dependence on cell cycle and oxygen concentration, and can trigger cell death through a variety of mechanisms, including apoptosis, necrosis, autophagy, premature aging, accelerated differentiation and/or delayed germ cell death. CIRT induced DNA damage is a mitotic catastrophe. Some scholars have contrasted the treatment of cancer cells with C ions and cisplatin, X-rays, and the results showed that mitotic catastrophe was triggered by CIRT (21). The authors suggested that aberrant mitosis and subsequent mitotic catastrophe resulted from less efficient repair of the more complex DSBs after CIRT. It can be seen that compared with ordinary photons/rays, the gene expression induced by carbon ions has a greater difference. Among them, genes related to cell metabolism, cell/organelle tissue, cell cycle, DNA damage and repair pathway were up-regulated or down-regulated. These changes, in turn, make carbon ion damage to DNA more lethal and lower the rate of DNA repair. Of course, DNA DSBs are the deadliest among them. This damage makes it difficult for DNA to repair itself and eventually leads to cell death.

### Advantages

**Figure 3:**
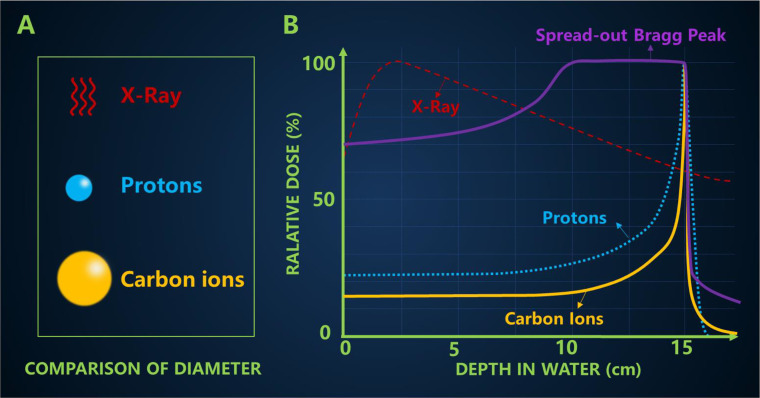
**(A)** Comparison of particle size **(B)** The typical depth dose curves of X-ray, protons and carbon ion beams, and a spread-out Bragg peak (SOBP) created by super-positioning many Bragg peaks at different depths.

Many cancer patients use X-rays, but when X-rays are delivered from an external source, they not only kill the target cancer cells, but they also damage healthy tissue by depositing most of their energy in it. X-ray is a kind of electromagnetic wave. The particle size of the carbon ion is significantly larger than that of the proton (**Figure 3A**).

Carbon ion irradiation has potential advantages over X-ray, including better physical dose distribution, greater reduction in lateral scattering (22), higher RBE and lower OER, all of which are ideal characteristics for killing radiation-resistant hypoxic tumors. The biggest difference between high-energy carbon ion beams and conventional X-rays is the depth dose curve, as shown in **Figure 3B**. The Bragg peak generated by carbon ions is sharper than that of protons, which may lead to a higher incident dose (23). In addition, a more pronounced “tail” can usually be observed behind the carbon ion range because nuclear interactions cause the carbon ions to break down into lighter particles (17). The penumbra of the high-energy proton beam is about 15 cm narrower than the penumbra of the X-ray beam. Compared with the former two qualities is heavier carbon ions, which makes carbon ion beam lateral scattering is smaller, and the corresponding penumbra area more acute, the closer the penumbra makes them maintain their direction when targeting tumor, further improve the accuracy of radiation, this will also help narrow pencil beam scanning control points better. In the longitudinal direction, carbon ions showed a more drastic dose drop than protons. The Bragg peak of the carbon ion beam enables it to provide most of the dose at the end of the range (Bragg peak), followed by almost no dose, leading to better normal tissue around the tumor area. However, because the tumor size is usually wider than the Bragg peak, the researchers developed different techniques to overlay the Bragg peak at different depths to form a SOBP that covers the entire tumor area and is evenly exposed to radiation. The carbon ions in SOBP energy can increase the energy of the body surface and the normal tissues in front of the tumor, but the energy of the normal tissues behind the tumor is still very low.

Osama believed that carbon ions caused cluster damage to DNA, both in terms of the high energy of carbon ions directly killing tumor cells and the complexity of indirect damage caused by ROS produced during the killing process, which made DNA repair difficult to achieve (6). Moreover, the higher the LET, the larger the DSBs, and the more the aggregation, and the more complex the DSBs, so as to promote carbon ions to cause a powerful lethal effect on cancer cells (24). A study from The University of Queensland has shown that CIRT induces more complex DSB, larger foci and more concentrated 53BP1 foci. Compared with x rays, CIRT caused greater differences in gene expression (25). CIRT can also induce irreparable DSBs, leading to increased cell killing from stem and non-stem cells (26) and from neuroblastoma and glioblastoma cell lines in patients with central nervous system (CNS) glioma. A study investigating the risk of secondary malignancy in prostate cancer patients found a lower risk of CIRT use due to reduced exposure to normal tissue using CIRT outside the target area (27). In addition, CIRT is one of the superior non-invasive methods for treating tumors in dangerous organs (such as heart and lung) and tumors resistant to conventional radiotherapy, while also allowing for combination therapy of target volumes, thereby increasing treatment rates. Clinical data of local recurrence of some gastrointestinal tumors, especially rectal cancer and pancreatic cancer, have been reported (28). **Table 2** provides a comparison of X-ray, proton, and carbon-based radiotherapy.

**Table 2:** Comparison between X-ray, proton, and carbon-based radiotherapy.

Parameter	Carbon Ions	Protons	X-ray
Year of First Treatment	1994	1954	Late 1800s and Early 1900s
Number of Sites Treating (Last Update: Sep 2020)	12	97	Routine
Worldwide treated patients, estimate	30,000^#^	190,000^#^	Millions
Volume of irradiated normal tissue	Smallest	Small	Large
Bragg-Peak	Present	Present	Absent
Estimated RBE	1.1-5.0	1.1	1.0
Relative LET	Highest	High	Low
Targeting precision	Highest	High	Low
In tumor tissue	High	Low	Low
In normal tissue	Low	Low	Low
Relative Risk of Secondary Malignancy	Low	Low	High
System cost	Highest	High	Low

^#^Dates from Particle Therapy Co-Operative Group.

### Shortcomings and Problems

#### The Technique Is Difficult and the Parameters Are Uncertain

Improving the biological effects of cancer therapy while reducing the dose to healthy tissue is a challenge. Compared to X-rays, beams of carbon ions are very sensitive to distance errors, which can lead to significant overdose or underdose. In addition, Bragg Peak is more likely to exist in a normal organization within a movement. The CIRT also shows a fragmented tail, causing greater uncertainty to the distal target tissue. Planning target volume (PTV) (suggested by ICRU report 50) has long been used to address the uncertainty of parameters during radiotherapy setting. The treatment plan of CIRT is designed based on the anatomical information obtained by the patient from the X-ray generated CT image. A margin of 3-6 mm is usually added to the clinical target volume (CTV) of PTV. However, this derivation is not accurate, and the error can reach 5 mm even within the range of 10 cm particles. For example, in 2016, Karasawa used carbon ions to treat head and neck cancer, where they added a 5mm margin to the CTV to ensure that the skin dose did not exceed 30 Gy (RBE) per week and was less than 50% of the prescribed dose (29). While, in 2017, Romeser used proton therapy for recurrent head and neck cancer increased the margin for PTV by 3 mm and added 10-20Gy (RBE) to the brain stem, spinal cord, and optic nerve structures, according to the interval since prior RT (30). In order to solve this uncertainty, people usually adopt optimized treatment regimens, such as setting the maximum tolerance value (31). However, when critical structures are very close to the target volume, consideration of these uncertainties may significantly affect the dose-shaping ability of using a carbon ion beam.

#### Lack of Data and Unclear Mechanism

Compared with X-ray treatment, CIRT treatment is still in the exploratory stage, with millions or more patients treated with X-ray, but only a few tens of thousands of cases treated with CIRT. As a result, the clinical data of CIRT is limited, and many problems in the treatment process are still to be solved. In addition, many of the biological mechanisms of carbon ion therapy remain uncertain. The exact extent and quality of the biological effects of carbon ions along its path, for example, remains uncertain. This uncertainty is caused by the special radiobiological properties of densely ionized heavy ion orbitals, which may be a problem of toxicity in normal tissues. At the same time, although the current reports do not find secondary cancer, but may have a long incubation period, this is still a problem to be considered. CIRT is rarely used, is still considered “experimental” for many tumor sites, and guidelines for clinical indications are still being established (11, 28). It is not yet possible to draw conclusions about its efficacy or toxicity, and further studies are needed to obtain more reliable data on its efficacy and toxicity.

#### High Cost, Difficult to Promote

Despite CIRT’s advantages and promising preclinical and clinical data to date, the cost of the carbon-based device is high due to the complexity of the system, such as the need for a synchrotron and additional shielding, and the cost-effectiveness of this expensive treatment remains unclear. So there are only a few institutions around the world that do CIRT. According to PTCOG, only 12 organizations are using carbon ions. The biggest obstacle to the widespread use of CIRT is the high initial investment in c ion centers and the high cost of maintenance and treatment, which makes it impossible for most hospitals and universities to set up c ion centers. Only a few studies have assessed the cost-effectiveness of CIRT.

## Clinical Experiences With CIRT

As mentioned above, C-ions have unique physical biological properties that have the potential to be ideal heavy particle candidates for cancer therapy. In addition to this, CIRT has significant capacity and potential to overcome DNA repair mechanisms. Moreover, many CIRT modalities involve hypo fractionation, which improves treatment efficiency and cost-effectiveness (32, 33). **Table 3** summarizes the current representative clinical trials based on CIRT therapy, including the related clinical data, including the organization, central location, types of cancer and primary end-point or results and so on. Clinical data obtained so far show that even tumors that are difficult to treat, such as those that are deep, critical, traditionally considered radiation-resistant, or recurrent and highly invasive, the results are reasonable (28).

**Table 3:** A list of selected open and/or recruiting clinical trials using CIRT alone or in combination with other treatment modalities.

No.	Organization, Central Location	Cancer	Trial Arms	Recruitment status	Target sample size	Primary end-point or Results
		Histology/Site				
1	NIRS, Japan	Pancreatic cancer	/	No longer recruiting	46	3-year overall survival
2		Liver tumor	/	No longer recruiting	50	Overall survival
3		Sinonasal and oral cavity cancers	/	Completed	60	Development of oronasal fistula
4		Malignant tumor of sphenoid sinus	/	Completed	20	Overall survival;
						Local control;
						Late toxicity
5		Recurrent head and neck tumors	/	Completed	48	The 2-year local control, loco regional control, progression-free survival, and overall survival rates were 40.5, 33.5%, 29.4%, and 59.6%, respectively.
6		Renal cell carcinoma	Single arm: Carbon ion (16 or 12 fractions)	Completed	27	Safety
7		The lacrimal gland carcinoma	/	Completed	33	Local control rates 62% (5 yr)
						Overall survival rates (65%)
8		Stage I non-small cell lung cancer	Single arm: Carbon ion (single fractions)	Unpublished	200	Local control;
						Overall survival
9		Mucosal Malignant Melanoma of the Head and Neck	/	Completed	100	Local control;
						Overall survival
10		Lung cancer or metastatic lung tumor	/	Completed	95	Toxicity;
						Local control rates 54% (2 yr);
						Overall survival rate 61.9% (2 yr)
11		Oral malignancies	Single arm: Carbon ion	Completed	83	Local control, progression free survival rates, and overall survival
12		Skull base and upper cervical spine chordoma	Single arm: Carbon ion	Completed	51	Local control
						Overall survival
13		Malignancy located chest or abdomen	Single arm: Carbon ion	Completed	10	Acute adverse reaction
14		Metastatic lung tumor	Single arm: Carbon ion (1-26 fractions)	Completed	100	Local control rates 79.9% (5 yr);
						Overall survival rate 58.9% (5 yr)
15		Solitary lymph node recurrence	Single arm: Carbon ion	Unpublished	310	2-year local control
						Incidence of grade 2 or worse late toxicities
16		Pancreatic cancer	Single arm: Carbon ion (12 fractions)	Open public recruiting	24	Rate of grade 3-5 acute toxicity
						Overall survival, local control
17		Kinds of cancer	/	Enrolling by invitation	999	Overall survival
18		Primary pancreatic cancer	Single arm: Carbon ion	Terminated	10	Acute normal tissue damages
19		Adenoid cystic carcinoma	Single arm: Carbon ion	Completed	100	Toxicities (acute and late)
						5-year overall survival
20		Mucosal malignant melanoma	Single arm: Carbon ion	Completed	20	5-year overall survival
21		Pelvic recurrent rectal cancer	Single arm: Carbon ion (16 fractions)	Open public recruiting	71	3-year overall survival
22		Malignant tumor located in thorax or abdomen	Single arm	Completed	12	Acute toxicity
						Initial response of local tumor
23		Breast cancer	Single arm: Carbon ion (4 fractions)	Open public recruiting	20	Acute toxicities of normal tissue
24		Malignant tumor	Single arm	Completed	15	Acute radiation toxicity
25		Head and neck cancer except sarcoma	Single arm: Carbon ion	Completed	1000	Overall survival
						Local control
26		Pancreatic Cancer	Single arm: Carbon ion (8 fractions)	Open public recruiting	10	Acute toxicity of organ at risks
27		Renal Cancer	Single arm: Carbon ion	/	10	Acute radiation toxicity of normal tissue
28		Prostate cancer	Single arm: Carbon ion (12 fractions)	No longer recruiting	45	Incidence of late radiation toxicity
29		Locally advanced adenocarcinoma of the uterine cervix	Single arm: Carbon (20 fractions)	Open public recruiting	32	Phase I study
						Acute toxicity
30	University of Texas Southwestern Medical Center, Department of Radiation Oncology Dallas, USA	Locally advanced, unresectable pancreatic cancer	Arm 1: Carbon ion	Completed	103	2-year overall survival
			Arm 2: Chemotherapy			
31		Locally Advanced Pancreatic Adenocarcinoma	Arm 1: Carbon ion	Active, not recruiting	110	2-year overall survival
			Arm 2: Photon			
32	Gunma University, Heavy Ion Medical Center Gunma, Japan	Prostate cancer	Single arm: Carbon ion (12 fractions)	No longer recruiting	300	Expanded prostate cancer index composite (epic)
33		Prostate, pancreatic, or uterine cancer	Single arm: Carbon ion	Completed	30	To evaluate tumor movement using CT images acquired on the treatment days and the treatment planning CT images.
34		Head and neck cancer	Single arm: Carbon ion	Open public recruiting	43	Dermatitis, microsites, QOL
35		Lung or Liver cancer	Single arm: Carbon ion	Open public recruiting	20	To evaluate tumor movement using CT images acquired on the treatment days and the treatment planning CT images.
36		Head and neck cancer	Single arm: Carbon ion	Preinitiation	40	Dermatitis/microsites
37		Locally advanced pancreatic cancer	Single arm: Carbon (12 fractions)	–	20	2-year overall survival
38		Hepatocellular carcinoma	Single arm: Carbon ion (4 fractions)	No longer recruiting	35	3-year local control
39		Hepatocellular carcinoma	Single arm: Carbon ion (4 or 12 fractions)	Open public recruiting	130	3-year overall survival
40		Hepatocellular carcinoma	/	Completed	250	Overall survival
41		Primary liver cancer	Single arm: Carbon ion (12 fractions)	Completed	6	Dose-limiting toxicity
42		Hepatocellular carcinoma	Single arm: Carbon ion (4 fractions)	Completed	3	Acute toxicity
43		Recurrent tumor in previously irradiated site	Single arm: Carbon ion	Open public recruiting	30	1-year local control
44		Refractory malignant tumor	Single arm: Carbon ion	Open public recruiting	50	1-year local control
45		Lymph-node recurrence of malignant tumors	Single arm: Carbon ion (12 fractions)	Open public recruiting	20	2-year local control
46		Malignant melanoma of head and neck	Single arm: Carbon ion (16 fractions) plus	Preinitiation	25	3-year overall survival and cause-specific survival rate
			Chemotherapy			
47		Clinical stage III non-small cell lung cancer	/	Open public recruiting	/	Acute adverse effect
48		Prostate cancer	Single arm: Carbon ion (16 fractions)	Completed	130	Biochemical relapse-free rate at 5 years
49		Clinical stage I non-small cell lung cancer	Single arm: Carbon ion (4 fractions)	Completed	40	The actuarial 2-year, 3-year, and 5-year local control rates were 91.2%, 88.1%, and 88.1%, respectively. The actuarial 2-year, 3-year, and 5-year overall survival rates were 91.9%, 80.0%, and 74.9%, respectively.
50		Pediatrics	Single arm: Carbon ion	Open public recruiting	6	Acute complication rate
51		Head and neck sarcoma	Single arm: Carbon ion	Open public recruiting	15	3-year local control
52		Primary skull base tumor	Single arm: Carbon ion 16 fractions)	Open public recruiting	20	3-year local control
53		Head and neck cancer	Single arm: Carbon ion	Open public recruiting	30	3-year local control
54	Kanagawa Cancer Center, Kanagawa Prefectural Hospital Organization, Ion-beam Radiation Oncology Center Kanagawa, Japan	Peripherally located stage-I non-small cell lung cancer	Single arm: Carbon ion (12-16 fractions)	Open public recruiting	162	Proportion of patients who developed glade 2 or early severe adverse events related to lung and skin
55		Locally advanced pancreatic cancer	Single arm: Carbon ion (12 fractions) plus	Open public recruiting	77	Overall survival;
			Chemotherapy			Local control
56		Patients with Prostate Cancer of Clinical Stage t1c-T3N0M0	Single arm: Carbon ion (12 fractions)	No longer recruiting	689	Biochemical progression-free survival at 5 years
57		Hepatocellular carcinoma	Single arm: Carbon ion (2 or 4 fractions)	Open public recruiting	50	3-year local control
58		Mucosal malignant melanoma of the head and neck	Single arm: Carbon ion (16 fractions) combined with anti-tumor agents	No longer recruiting	65	3-year overall survival
59		Small-sized peripheral non-small cell lung cancer with clinical stage IA	Arm 1: Carbon ion	Open public recruiting	525	5-year overall survival
			Arm 2: Surgical resection			
60		Non-squamous cell carcinoma of head and neck	Single arm: Carbon ion (16 fractions)	No longer recruiting	54	3-year local control
61		Hepatocellular Carcinoma	Single arm: Carbon ion (2 or 4 fractions)	Open public recruiting	50	3-year local control
62		Localized prostate cancer	Single arm: Carbon ion (12 fractions)	No longer recruiting	145	Biochemical progression-free survival at 5 years
63		Localized prostate cancer	Single arm: Carbon ion (12 fractions)	No longer recruiting	145	Biochemical progression-free survival at 5 years
64		Localized or locally advanced prostate cancer	/	No longer recruiting	12	Incidence of late-phase rectal adverse event with CTCAE Grade 2 or more
65	Jichi Medical University, Department of Radiology Tochigi Japan	Lymph node recurrence of gynecological cancers	Single arm: Carbon ion	Unpublished	15	2-year overall survival
66	Ion Beam Therapy Center, SAGA-HIMAT Foundation, Department of Radiation Oncology, Tosu, Japan	Peripherally located inoperable stage-I non-small-cell lung cancer	Single arm: Carbon ion (4 fractions)	Open public recruiting	150	3-year overall survival
67		Centrally located stage I non-small-cell lung cancer	Single arm: Carbon ion (12 fractions)	Open public recruiting	20	3-year local control
68		Peripherally located stage I non-small-cell lung cancer	Single arm: Carbon ion (4 fractions)	Open public recruiting	65	3-year local control
69		Hepatocellular carcinoma	Single arm: Carbon ion (2 fractions)	No longer recruiting	35	3-year local control
70	University Hospital Heidelberg, Juergen Debus	Skull Base Meningioma	Four arms: Carbon ion (15 fractions)	Not yet recruiting	80	No results posted
			Proton (15 fractions);			
			Hypo fractionated Photon (15 fractions);			
			Conventional Photon (32 fractions);			
71		Locally Advanced Pancreatic Cancer	Single arm: Carbon ion	Interventional (Clinical Trial)	0	Acute toxicity of carbon ion radiotherapy observed within 3 months of study treatment.
72		Prostatic Neoplasms	Arm 1: Proton;	Completed	92	Proctitis and cystitis *via* incidence grade 3-4 toxicity
			Arm 2: Carbon ion			
73		Recurrent Rectal Cancer	Single arm: Carbon ion	Completed	14	Safety and Efficacy
74		Chordoma	Arm 1: Carbon	Recruiting	319	8-year Local-Progression Free Survival
			(15 fractions); Arm 2: Proton			
75		Chondrosarcoma	Arm 1: Carbon ion	Recruiting	154	5-year Local-Progression Free Survival
			(15 fractions); Arm 2:Proton			
76		Glioma	Single arm: Carbon ion (10-16 fractions)	Completed	56	No results posted
77		Primary Glioblastoma	Arm 1: Carbon ion (6 fractions); Arm 2: Proton	Completed	100	1-year overall survival
78	Shanghai Proton and Heavy Ion Center, China	Recurrent Nasopharyngeal Carcinoma	Single arm: Carbon ion	Not yet recruiting	40	No results posted
79		Hepatocellular Carcinoma	Single arm: Carbon ion (5 fractions)	Withdrawn (enrollment was too slow)	0	Progression-free survival of all patients
80		Adenoid Cystic Carcinoma	Arm 1: Carbon ion; Arm 2: Proton	Recruiting	50	No results posted
81		Metastatic Prostate Carcinoma	Carbon ion radiotherapy combined with systemic therapy	Recruiting	47	Time to PSA relapse
82		Hepatocellular Carcinoma	Single arm: Carbon ion (10 fractions)	Recruiting	48	Number of participants with treatment-related adverse events as assessed by CTCAE v4.0
83		Nasopharyngeal Carcinoma	Arm 1: Carbon ion	Terminated (Slow accrual of patients.)	9	No results posted
			Arm 2: chemotherapy			
84		Nasopharyngeal Carcinoma	Single arm: Carbon ion	Active, not recruiting	55	No results posted
85		Prostate Carcinoma	Single arm: Carbon ion (16 fractions)	Recruiting	61	Number of participants with treatment-related adverse events as assessed by CTCAE v4.0
86		Nasopharyngeal Carcinoma	Single arm: Carbon ion	Terminated (Slow accrual of patients.)	9	Number of participants with treatment-related adverse events as assessed by CTCAE v4.0
87	Albert Einstein College of Medicine, Nitin Ohri	Pancreatic Cancer	Arm 1: Carbon ion	Completed	14	Dose-limiting toxicity
						Any CTCAE v. 4.03 non-hematologic adverse event of grade 3 or higher or any hematologic adverse event of grade 4 or higher, occurring within 90 days of the start of radiotherapy and deemed to be related to carbon ion radiotherapy.
88	European Institute of Oncology	Adenocarcinoma of Prostate	Arm 1: Carbon ion; Arm 2:	Recruiting	65	There were level 3 or level 4 adverse events
			Proton			
89	Hospices Civils de Lyon	Malignant Tumors as Chordoma, Adenoid Cystic Carcinoma and Sarcoma	Arm 1: Carbon ion; Arm 2:	Recruiting	250	5-year Progression free survival
			X-rays and/or protons			

This information of the list comes from information available www.umin.ac.jp/ctr and https://ptcog.ch. UMIN, University hospital Medical Information Network; NIRS, National Institute of Radiological Sciences.

**Figure 4:**
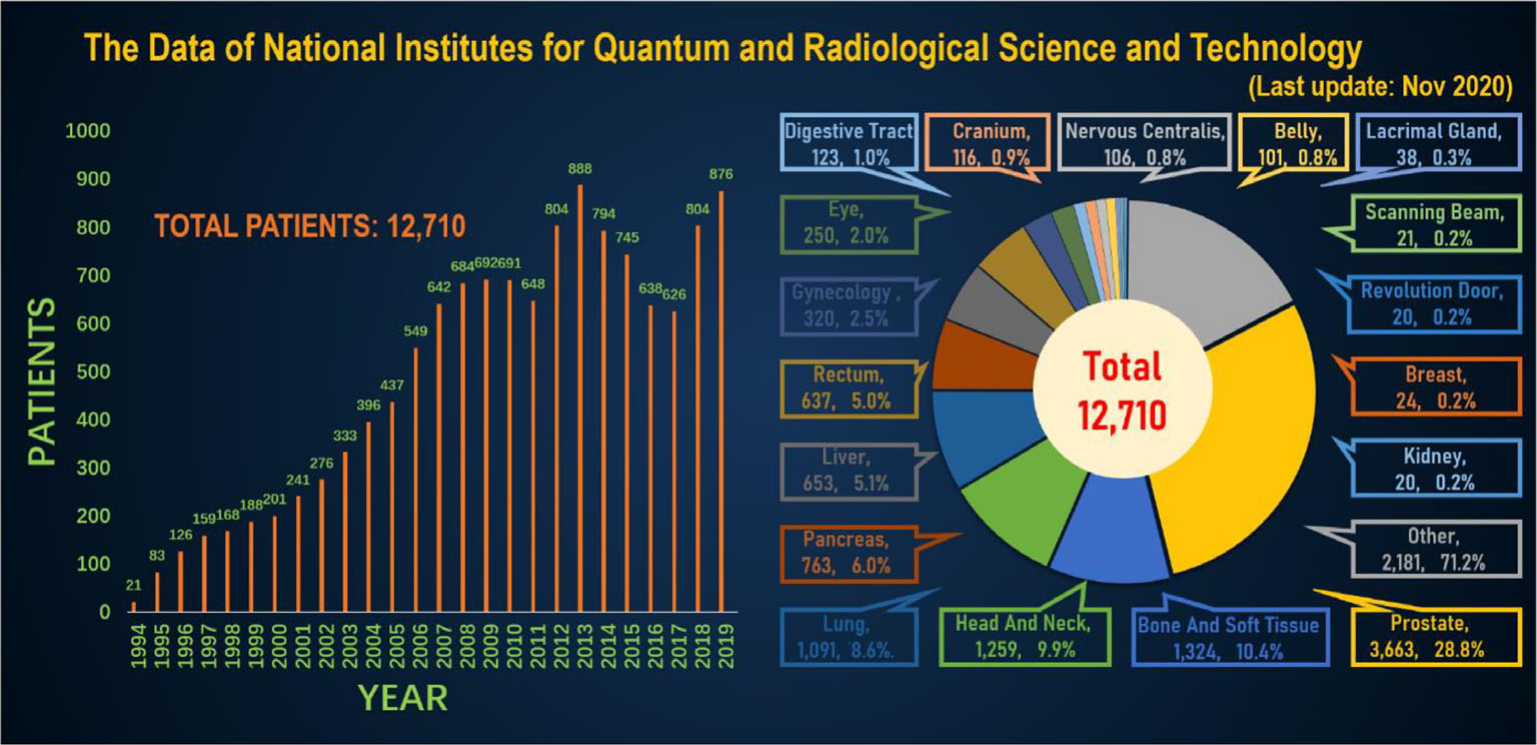
The annual number of patients treated with heavy ions and the distribution of heavy ion radiotherapy in Japan’s NIRS for various cancer patients.

**Figure 5:**
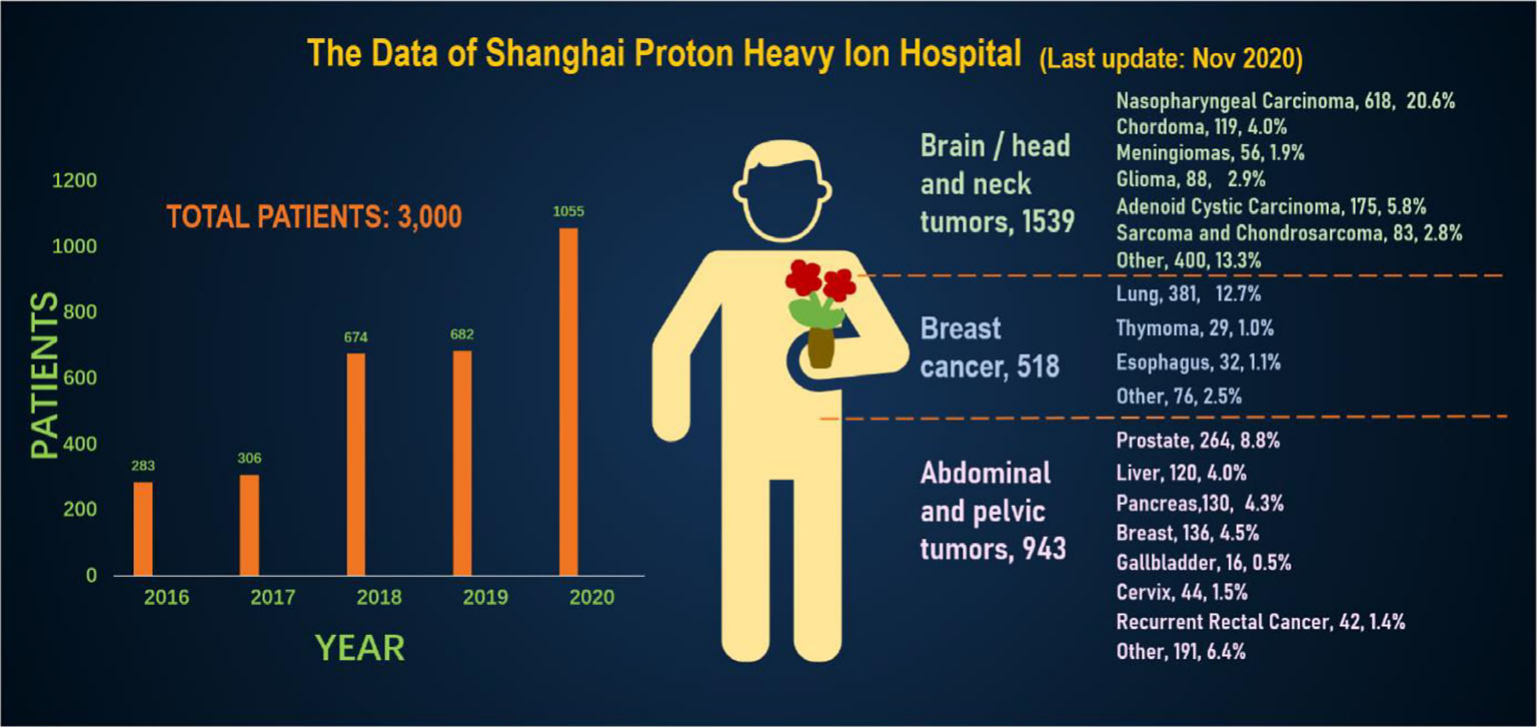
The annual number of patients treated with heavy ion therapy alone or proton therapy alone or heavy ion combined with proton therapy in Shanghai Proton Heavy Ion Hospital for various cancer patients.

In order to better and more intuitively understand the current status of heavy ion therapy, we made statistics on the data of National Institutes for Quantum and Radiological Science and Technology and Shanghai Proton Heavy Ion Hospital, respectively. According to relevant data of the National Institutes for Quantum and Radiological Science and Technology (website: www.qst.go.jp.) relevant data, in which there were about 11,834 patients who using CIRT alone or in combination with other treatment modalities, from the start of clinical trials in June 1994 to March 2019. The annual number of patients treated with heavy ions and the distribution of heavy ion radiotherapy in Japan’s NIRS for various cancer patients, and among the admitted patients, prostate cancer was 27.8%, bone and soft tissue tumors 10.7%, head and neck cancer 10.1%, lung cancer 8.9%, pancreatic cancer 5.9%, Liver cancer was 5.2%, rectal cancer was 5.1% (**Figure 4**). The annual number of patients treated with heavy ion therapy alone or PT alone or heavy ion combined with PT in Shanghai Proton Heavy Ion Hospital for various cancer patients. By September 30, 2020, Shanghai Proton Heavy Ion Hospital has treated more than 3,000 discharged patients, with an average annual growth rate of 28% (website: www.sphic.org.cn). Among them, a total of 2836 patients were treated with heavy ion therapy alone or combined with heavy ion proton therapy (1083 cases with heavy ion therapy, 1753 cases with heavy ion combined with PT), accounting for 94.5%, making full use of and giving full play to the advantages of heavy ion therapy technology. Recently, the treatment volume of monomer heavy ion in this treatment facility has been maintained at about 80 treatments, and the preparation time before admission has been shortened by 10.4 days. Among the 3,000 patients who had been treated and discharged from hospital, 1,539 cases were head and neck tumors, including 618 cases of nasopharyngeal cancer, 119 cases of chordoma, 56 cases of meningioma, 88 cases of glioma, 175 cases of adenoid cystic carcinoma, 83 cases of sarcoma and chondrosarcoma, and 400 other cases. There were 518 cases of breast tumor, including 381 cases of lung cancer, 32 cases of esophageal cancer, 29 cases of thymic carcinoma and 76 other cases. There were 943 cases of tumors in the abdomen and pelvis and other areas, including 264 cases of prostate cancer, 120 cases of liver cancer, 130 cases of pancreatic cancer, 136 cases of breast cancer, 16 cases of gallbladder, 44 cases of cervical cancer, 42 cases of recur rectal cancer after surgery, and 191 other cases. Guided by patient needs, Shanghai Proton Heavy Ion Hospital conducts clinical treatment centering on five key diseases with the highest incidence in China, including nasopharyngeal cancer, cranial base tumor, lung cancer, liver cancer and prostate cancer, and conducts key clinical research on pancreatic cancer, with the number of patients with key diseases accounting for 64% of the total (**Figure 5**). A summary of selected clinical outcomes for treatment of kinds of cancers with CIRT is in **Table 4**. It can be seen that CIRT is effective and safe for most cancers, and is less toxic.

**Table 4:** A summary of selected clinical outcomes for treatment of kinds of cancers with C-ion therapy.

Cancer	Patient number	Target dose, GyE	Overall survival	Local control	Late toxicity at reporting and results
Recurrent NPC	75	50-60	98.1% (1 yr)	LRFS 86.6% (1 yr); RRFS 97.9% (1 yr)	7 necrosis at tumor bed, including 1 carotid blowout (34).
STS	57	52.8-73.6	82% (1yr)46% (3 yr)	88% (1 yr); 73% (3 yr)	Without grade > 3 acute reactions. Effective and safe (35).
	24	52.8-73.6	75% (2 yr)	77% (2 yr); 69% (5 yr)	No other toxicity greater than Grade 2 was observed. Effective and safe (36).
			50% (5 yr)		
	78	70.4	33% (5 yr)	62% (5 yr)	Toxicity occurs in individual patients. Effective and safe (37).
	17	52.8-70.4	56% (5 yr)	76% (5 yr)	Toxicity (grade 3) was not observed in most patients. Effective and safe (38).
	47	52.8-70.4	52% (5 yr)	79% (5 yr)	Without fatal toxicities (39).
	188	64-73.6	81%(5 yr)	77% (5 yr)	Toxicity occurs in individual patients. Effective and safe (40).
	75	57.6-73.6	57% (5 yr)	55% (5 yr)	Toxicity occurs in individual patients. Effective and safe (41).
Head and Neck	236	57.6-64.0	35-68% (5 yr)	24-75% (5 yr)	Promising outcomes with reduced acute and late reactions.
					Effective and safe (42).
	53	24	78% (3 yr)	82% (3 yr)	Acceptable toxicity. Treatment was tolerated, with moderate acute and late toxicity (43).
Adenoid cystic carcinomas	18	57.6-67.4	72% (5 yr)	92% (5 yr)	Regarding late reactions, 2 patients developed grade 3 mandible osteoradionecrosis, and 1 had grade 3 hemorrhage of the tongue base. Effective and safe (44).
	309	23.9	88.9% (3yr)	83.7%(3 yr)	only 4% of patients developed grade III dysphagia and late toxicities of grade 3 or higher occurred in only3 patients (45).
			74.6% (5yr)	58.5% (5 yr)	
	289	55.2-70.4	94% (2 yr)	88% (2 yr)	15% of the patients experienced late toxicities that were scored as grade 3 or higher, with osteonecrosis
			74% (5 yr)	68% (5 yr)	being the most common (46).
	58	18	76.5% (5 yr)	59.6% (5 yr)	C12 therapy resulted in superior LC, PFS, and OS without a significant difference between patients with inoperable and partially resected ACC (47).
Mucosal melanomas	18	74	16.2% (3 yr)	58.3 (3 yr)	Grade III or higher late toxicity was not observed. CIRT has shown good local control in mucosal melanomas but long-term survival is still poor (48).
Choroidal melanoma	116	60-85	80.4% (5 yr)	92.8% (5 yr)	The long term outcomes of CIRT for choroidal melanoma with excellent local control and eye retention rates (49).
	79	60	98% (5 yr)	89% (5 yr)	Acute and late toxicities were mild. With no grade > 3 reactions. Safe and effective (50).
			79% (10 yr)	88% (10 yr)	
Chordomas	96	60	88% (5 yr)	70% (5 yr)	Acute and late toxicities were mild. With no grade > 3 reactions. Safe and effective (51).
			75% (10 yr)	54% (10 yr)	
Skull base chordoma	155	60–74	95% (3 yr)	82% (3 yr)	safe and effective (52).
			85% (5 yr)	72% (5 yr)	
			75% (10 yr)	54% (10 yr)	
	33	48–60.8	87.7% (5 yr)	85.1% (5 yr)	Normal tissues showed a mild reaction without any severe morbidity of important organs (53).
			67% (10 yr)	63.8% (10 yr)	
	23	70.4	83% (3 yr)	94% (3 yr)	Toxicity (≥ grade 3) late were observed in nine patients. Useful and safe (54).
	188	64–73.6	81.1% (5 yr)	77.2% (5 yr)	Toxicity occurs in individual patients. Effective and safe (40).
	56	60–74	100% (2 yr)	76% (2 yr)	No higher toxicity occurred within the follow-up time. Effective and safe (55).
				53% (3 yr)	
skull base chondrosarcomas	101	60	100% (1 yr)	98.6% (1 yr)	No toxicity worse than Common Toxicity Criteria grade 3 was observed after treatment (56).
			98.5% (2 yr)	97.2% (2 yr)	
			92.9% (4 yr)	90.5% (4 yr)	
Skull Base Sarcomas	53	54-73.5	91.2% (1 yr)	LRFS: 89.2% (1 yr)	With few observed acute and late toxicities. Safe and effective (57).
			80.2% (2 yr)	80.2% (2 yr)	
meningioma	42	36–60	89.6% (1 yr)	71% (1 yr)	Safe and effective (58).
			71.4% (2 yr)	56.5% (2 yr)	
Prostate Cancers	175	66	/	/	No grade *≥* 3 toxicities (59).
	46	51.6-57.6	/	/	No other G2 acute toxicities were observed. The new shortened CIRT schedule over 3 weeks was considered as feasible (60).
	664	57.6	95.2% (5 yr)	/	Advancement in hypofractionation could be safely achieved with C-ion RT for prostate cancer (61).
	2157	51.76-66	96-100(5 yr)	96-99% (5 yr)	No grade *≥* 3 toxicities. Favorable overall outcomes of CIRT for prostate cancer (62).
LACC	22	64-72	50% (5 yr)	68.2% (5 yr)	No grade 2 toxicities. CIRT has the potential to improve the treatment for locally YOUYIJIANadvanced bulky cervical cancer (63).
HCC	64	52.8	22 (5 yr)	88 (5 yr)	Excellent local control was obtained independent of tumor location (64).
	76/58	55.2	48% (2 yr)	83% (2 yr)	Safe and effective (65).
	24	49.5-79.5	92% (1 yr)	92% (1 yr)	Safe and effective (66).
			50% (3 yr)	81% (3 yr)	
			25% (5 yr)	81% (5 yr)	
	126	48-54	90% (1 yr)	95% (1 yr)	Safe and effective (67).
			50% (3 yr)	91% (3 yr)	
			25 (5 yr)	90 (5 yr)	
	101	52.8-76	36% (5 yr)	93% (5 yr)	Safe and effective (68).
	31	52.8-60	82% (2 yr)	89% (2 yr)	C-ion RT was effective with minimal toxicities for 80 years or older patients with hepatocellular carcinoma (69).
Pancreatic Cancers	26	30-36.8	42% (5 yr)	None of the patients experienced local failure.	Safe and effective (70).
	64	55.2	84% (1 yr)	82% (2 yr)	No grade *≥* 3 toxicities (71).
			53% (2 yr)		
	72	52.8-55.2	73% (1 yr)	/	No patients developed late grade 4 or 5 toxicity (72).
			46% (2 yr)		
	72	43.2–55.2	73% (1 yr)	/	Carbon ion RT with concurrent full-dose gemcitabine was well tolerated and effective in patients with unresectable locally advanced pancreatic cancer (65).
			48% (2 yr)		
Recurrent and Previously Irradiated Cancers	52	67-182	/	70.3% (1.2 yr)	Without grade > 2 toxicity. Further dose escalation should be viewed with caution (73).
Recurrent Rectal Cancer	180	73.6	59% (5 yr)	88% (5 yr)	Without grade > 3 toxicities. Safe and effective (74).
sinonasal malignancies	911	18-24	75.1% (3 yr)	80.2% (3 yr)	Safe and effective (75).
Breast Cancer	7	48-60	All cases were alive without recurrence (5 yr)		At the end of 2017, all cases were alive without recurrence or late had not caused any late adverse reaction. Safe and effective (76).
	1	36	Surviving more than 8 years without local recurrence.		Safe and effective (77).

Recurrent NPC, Recurrent nasopharyngeal carcinoma; STS, Osteosarcomas and Soft Tissue Sarcomas; HCC, Hepatocellular Carcinomas; LACC, Locally Advanced Cervical Cancer; LRFS, Local Relapse Free Survival; RRFS, Regional Relapse Free Survival.

## Sensitizing Agent

CIRT therapy has significant advantages over other approaches to treating cancer, but it still has a lot of room for improvement. Meanwhile, Increasing the maximum dose accumulation in tumor tissues while also looking to reduce the damage to normal tissues has always been a great challenge in radiotherapy. Different treatment strategies have been proposed to balance treatment outcomes with side effects, such as reversing radiation resistance of tumor tissue, increasing radiation sensitization of tumor tissue, and limiting the deposition of radiation dose in tumor volume (78). Current efforts are being made to achieve the goal of improving the biological effects of carbon ion irradiation by using approaches such as cellular pathway inhibitors (79), small chemical drugs (80, 81), and metallic nanomaterials (82, 83). Among them, sensitized agents (usually nanomaterials) refer to chemical or biological compounds that enhance the effective dose of RT on cancer cells, either through increased permeability and retention effects or through the use of targeted biomolecules that accumulate in tumors. It has been developed as a nano-enhancer to increase the physical irradiation dose of biological effects, which has attracted wide attention (84, 85).

Due to the high cost of carbocation therapy, its application is few, and the related sensitizing agent research is very rare. According to research, only a few metal nanoparticles have been used to improve carbon ion irradiation. In this chapter, we combined existing reports and sensitizers commonly used in radiotherapy to review the research on carbon ion sensitizing agent, in order to provide some reference and inspiration for relevant researchers.

### Common Sensitizing Agent

Chemotherapy sensitizing agents were the first group of sensitizing agents on the treatment of cancer. They were often combined with chemotherapy drugs to increase the effectiveness of chemotherapy. Common chemotherapy drugs are gemcitabine, docetaxel, pemetrexed, paclitaxel, and some platinums, including cisplatin, carboplatin, and some nedaplatin. While, due to the drug resistance of some tumors, chemotherapy drugs cannot successfully achieve the expected effect of tumor treatment, so some combination therapy through chemotherapeutic sensitizing agent [such as, lonidamine (86–90), poloxamers, silibinin (91)], immune checkpoint inhibitors [such as PD-1 inhibitors, PD-L1 inhibitors (92)], enzyme related inhibitors [such as ribonucleotide reductase inhibitors, crizotinib (93)], as well as other tumor-related inhibitors have become a research hotspot.

High atomic element (Z) nanomaterials, such as bismuth (Z=83), gold (Z=79), tungsten (Z=74), tantalum (Z=73), hafnium (Z=72), tellurium (Z=52), silver (Z=47), are capable of increasing the production of secondary and Auger electrons, which in turn increases the generated ROS and enhances the deposition of radiation. These elements are also called “nano enhancers” and they have much higher mass-energy absorption coefficients than soft tissues (94, 95). Therefore, nanoparticles based on high altitude subordinal metal as ionizing radiation sensitizing materials are getting more and more attention.

Some of them also play a role in the treatment of cancer as the preparation nanomaterials of relatively common chemotherapeutic sensitizing agents, immune checkpoint inhibitors, enzyme-related inhibitors and tumor-related inhibitors, and some of them cooperate with other therapies as their sensitizing agents to achieve anti-cancer. In 2004, Hainfeld (96) reported for the first time that gold (Z=79) nanoparticles could enhance the effect of tumor radiotherapy and inhibit tumor growth *in vivo*. Zhang (97) prepared gold nanoparticles coated with glutathione and composed of several gold atoms, which also confirmed this point. The author attributed its excellent targeting ability to good biocompatibility and ultra-small particle size, and the results of tumor inhibition experiments also confirmed that “gold nanomolecules” had a good radiotherapy sensitization effect. Wang (98) constructed a kind of mesoporous silica (SiO_2_) coated Janus gold nanorods. The unique structure of the material can on the one hand deposit the radiotherapy sensitization effect of the nanorods, and on the other hand can realize the efficient loading of doxorubicin as a chemotherapy drug. Chen designed a kind of Au@Se-R/A nanocomposite (Au@Se-R/A NCs) based on the radiotherapy sensitizer properties of gold nanorodes (NRs) and the antitumor activity of selenium NPs to realize synergistic chemoradiotherapy (99). Moreover, *in vitro* studies showed that the combined treatment of NCs and X-ray in A375 melanoma cells could significantly improve the anti-cancer efficacy by changing the expression of p53 and DNA damage genes, inducing cell apoptosis and triggering the excessive production of intracellular ROS. Subsequently, they synthesized a tellurium (Te, Z=52) nanosar (GTe-RGD), providing a therapeutic strategy that combines GTe-RGD-enhanced RT with checkpoint blockade immunotherapy to effectively and systematically eliminate tumors, providing an attractive clinical alternative to oncology therapy (100). Duo designed a radiosensitizer of ultra-thin antimonene nanoparticles (AMNPs) that could achieve effective radio-chemotherapy effects by inducing a strong oxidative stress response and their significant high radiotoxicity. This technique could expand the application range of antimonene as an effective radiosensitizer, and promoted its modulated and effective radiosensitizer effect in clinic (101).

Gadolinium-based nanoparticles (GdNPs), which process high relaxation time and high atomic number (Z=64), have attracted substantial attention. GdNPs have high electron density. GdNPs have high electron density. Therefore, ionizing radiation contains notes nanoparticles aqueous solution, in addition to the interaction between ionizing radiation and water can cause water molecules ionization and secondary electron emission, the incident particles and secondary electron interaction with Gd will also lead to request for nanoparticles electron emission increases around a few nanometer scale, electronic further solution of released from water molecules lead to the water oxide increased ROS. The measurement of hydroxyl radicals, which play a major role in ROS, can directly reflect the sensitization effect of Gd on ionizing radiation. It is worth mentioning that GdNPs which as a T1-enhanced clinical magnetic resonance imaging material, can provide high-resolution clear imaging of soft tissues. GdNPs can be used as sensitizing materials for ionizing radiation, enhancing both X-ray and particle beam irradiation (102). Ultra-small gadolinium oxide nanocrystals (GONs) are attractive gadolinium nanocrystals, which have a high density of gadolinium/contrast agent units. GONs have been developed as advanced T1-weighted MRI contrast agents due to their high longitudinal relaxivities and small r2/r1 ratios. MA found that GONs have good biocompatibility in breast cancer McF-7 cells (103). Amirrashedi et al. first studied the radio sensitizing agent properties of GONs in a gel-filled volume model, where the maximum dose enhancement range of GONs was 15%-24% (104). Some scholars found that under photon and proton irradiation, the enhancement of ROS produced by GONs was dose-dependent, and the factor was 1.94 compared with radiation control alone. Core-inner-valence ionization of atoms could de-excite electrons by means of a powerful Gd-Gd interatomic de-excitation process. The radiosensitizing biomechanism of GONs under X-ray remains unclear. Shady Kotb et al. reported a kind of Gd-based nanoparticles AGuIX, which is composed of a polysiloxane core and its surrounding covalently attached gadolinium chelate network and with a hydrodynamic diameter of less than 5 nm. They concluded that AGuIX is an effective T1-MRI contrast agent, and that the combination of AGuIX and radiation not only significantly enhanced the dose *in vitro*, but also improved survival in mice with aggressive brain tumors, demonstrating the safety and efficacy of AGuIX as a potential clinical contrast agent and radiosensitizer (102).

Among all kinds of nanomaterials applied in radiotherapy, gold nanoparticles (GNP) has been considered as a potential tool for diagnosis and treatment of various cancers due to its small particle size, good dispersion, strong stability and good biocompatibility (105, 106). So GNPs have long been considered as a potential tool for the diagnosis and treatment of many types of cancer. GNPs are the first metal nanoparticles used in radiation sensitization research and they are also the most studied nanoparticles at present, and the enhancement effects of which have been demonstrated both *in vitro* and *in vivo (* 84, 94). In addition, some researchers have also reported some other sensitizers, such as silver nanoparticles (107), titanium oxide (108), bismuth oxide (109), etc.

### CIRT Sensitizing Agent

Although the materials mentioned above can be used as sensitizing agent for XRT and PT (110), few of them can be used as sensitizing agent for carbon ion therapy. Kaur found that the presence of glucose-capped gold nanoparticles in HeLa cells led to an enhancement of 41% in the RBE value of carbon ion irradiation (111). It has been reported that nano gold can significantly affect carbon ion irradiation, and this effect is obviously dependent on the concentration of nano gold. Porcel’s team found that Pt nanoparticles can significantly enhance DSB damage induced by carbon ion irradiation (112). What’s more, in another study, he found that gadolinium based nanoparticles (GBNPs) can enhance the sensitivity of Chinese hamster ovary cells to C^6+^ and He^2+^ radiation (113). In addition, AGuIX could significantly enhance the killing effect of carbon ions on head and neck anti-radiation tumor cells (83). In carbon ion therapy, more efforts are needed to expand the uses of metal nanoparticles and explore their biological mechanisms, especially for therapeutic agents such as gadolinium. Some researchers have studied the radiation sensitization effect of theranostic metal-based nanoparticles in carbon ion irradiation and its mechanism (94). Li found that pretreatment with GONs led to the enhancement of hydroxyl radical and ROS production, which contributes to cell cycle arrest at G2/M phase to allow for repair of damaged DNA with DSBs. They thought that based on the good biocompatibility, the instinctive advantage of Gd as an MRI contrast agent, and the sensitization effect stated above, GONs may be a potential theranostic sensitizer in NSCLC patients under carbon ion radiotherapy after further *in vivo* preclinical studies (114).

## Prospects and Concluding Remarks

With the aggravation of the aging of the global population and the acceleration of industrialization and urbanization, malignant tumors continue to grow globally and bring enormous physical, emotional and economic pressure to individuals, families, communities and health systems. It has become one of the major problems that seriously threaten human health. There is no doubt that the emergence of CIRT has brought good news to human health, especially for those recurrent cancer, anti-radiation cancer. In the case that increasing the radiation dose still fails to achieve good results, the emergence of CIRT undoubtedly provides a new opportunity. Not only are carbon ions able to kill tumor cells with greater precision, they also retain the most of healthy tissue. Another potential benefit of CIRT may be in combination with immunotherapy. CIRT with high LET radiation has been shown to have higher immunogenicity in radiation-induced cell death and therefore has a significant advantage in combination immunotherapy (115). The existing clinical pre-studies and on-the-spot studies have achieved remarkable success. But this new processing technology also inevitably brings some problems and troubles.

First of all, basic biological research of CIRT needs to be further explored. From the analysis of clinical data, there is no doubt about the effectiveness of CIRT for cancer, especially in the treatment of some recurrent cancer has been a great success. However, due to the high investment and related uncertainties in the aspects of carbocation dose transfer and radiobiological effects, it is unlikely to replace traditional radiotherapy as the mainstream treatment in the short term. The clinical trials related to CIRT need to be further strengthened, and more prospective trials are needed to clarify the role of CIRT in clinical practice. Secondly, the miniaturization of the CIRT device. The technology of CIRT is advanced, however the processing and operation of CIRT equipment requires high precision, large volume of equipment, and high requirements for the research technology of the accelerator. The miniaturization of the accelerator is still an important issue at present. Thirdly, the dynamic monitoring of tumor movement in radiotherapy is still an urgent problem to be solved at present to improve the effect of radiotherapy. Dynamically monitor tumor motion during RT mainly includes two parts: tumor detection and tracking model establishment. CIRT uses CT simulation to personalize the radiotherapy plan for the patient. CT simulation location is a virtual simulation process of target determination and planning based on CT images and other medical images, which is a kind of three-dimensional image simulation. After the completion of the CT, the physical and dosimetric experts will set the radiotherapy dose according to the scope outlined by the clinical experts, and design and complete the radiotherapy plan. So there is still some margin of error in the range that this calculation outlines. As for the establishment of tracking model, it involves many links of tumor signal acquisition, measurement, calculation, transmission, control and treatment. Therefore, it is difficult for CIRT to dynamically monitor tumor movement at present. In addition, compared with X-ray treatment, CIRT related sensitizers are still in the minority. The extreme sensitivity of the carbon ion beam leads to the uncertainty of the range of the carbon ion beam, which provides a new choice for the treatment of tumors and to a certain extent enhances the killing degree of tumor cells. However, due to the scarcity of carbon ion research and the lack of specific biological mechanism, the corresponding sensitizer is still in the preliminary stage of exploration. At present, only gadolinium-based, gold-based and other nanomaterials are known. The clinical application of carbon sensitizers remains challenging. Most notably, the construction cost of carbon ion treatment equipment and treatment cost need to be reduced. The investment cost of carbon ion therapy center is huge, and the late maintenance cost is also very expensive. With the high treatment cost borne by patients, the advantages of CIRT are greatly reduced in the face of further promotion and application. Therefore, reducing the cost of CIRT is still an important issue for people.

At present, more and more carbon ion centers are being prepared for construction around the world, prospective clinical trials on the treatment of various types of cancers are also being carried out gradually, and the technology of CIRT has become increasingly mature. Objective evaluation of the real advantages of CIRT, clear the value and limitations of carbon ion application, the use of existing conditions to do the basic data and basic technical research work, make full technical reserve, accumulate rich clinical experience. We believe that in the near future, with the progress of science and technology and the in-depth research, whether it is the reduction of equipment cost, treatment cost reduction, or the further conquest of cancer, the future will be gradually solved. The data of CIRT will be gradually improved, so that its characteristics and advantages can be more clearly understood, so as to provide more and more effective treatment options for cancer patients.

## Data Availability Statement

The raw data supporting the conclusions of this article will be made available by the authors, without undue reservation.

## Author Contributions

XW conducted the literature review and drafted the manuscript. TG, WL, and XT contributed to review and edited and formatted the final manuscript. XC, GL, and XH edited and contributed to the revised manuscript. All authors contributed to the article and approved the submitted version.

## Funding

The authors acknowledge funding from National Key R&D Program of China (2020YFC2007301, 2020YFC2007300).

## Conflict of Interest

The authors declare that the research was conducted in the absence of any commercial or financial relationships that could be construed as a potential conflict of interest.

## Abbreviations

CIRT, Carbon Ion Radiation Therapy; RT, Radiation Therapy; NCDs, Noncommunicable Diseases; WHO, World Health Organization; LBNL, Lawrence Berkeley National Laboratory; HIMAC, Heavy Ion Medical Accelerator; NIRS, National Institute of Radiological Science; HIRFL, Lanzhou Heavy Ion Research Facility; HIT, Heidelberg Ion-beam Therapy Center; PTCOG, Proton Therapy Co-Operative Group; SOBP, Spread-out Bragg peak; PET, Positron Emission Tomography; LET, Linear Energy Transfer; RBE, Relative Biological Effects; SSBs, Single Strand Breaks; DSBs, Double Strand Breaks; ROS, Reactive Oxygen Species; OER, Oxygen Enhancement Ratio; CNS, Central Nervous System; PTV, Planning Target Volume; CTV, Clinical Target Volume; GdNPs, Gadolinium-based Nanoparticles; GONs, Gadolinium Oxide Nanocrystals; GNP, Gold Nanoparticles; GBNPs, Gadolinium Based Nanoparticles; UMIN, University Hospital Medical Information Network; NPC, Nasopharyngeal Carcinoma; STS, Osteosarcomas and Soft Tissue Sarcomas; HCC, Hepatocellular Carcinomas; LACC, Locally Advanced Cervical Cancer; LRFS, Local Relapse Free Survival; RRFS, Regional Relapse Free Survival.
